# Thumb Interphalangeal Joint Dislocation

**Published:** 2016-01-11

**Authors:** Darnell J. Brown, Alexis L. Parcells, Mark S. Granick

**Affiliations:** Rutgers Robert Wood Johnson Medical School, New Brunswick, NJ

**Keywords:** thumb dislocation, thumb interphalangeal (IP) joint dislocation, IP dislocation of thumb, thumb IPJ dislocation, open thumb IPJ dislocation

**Figure:**
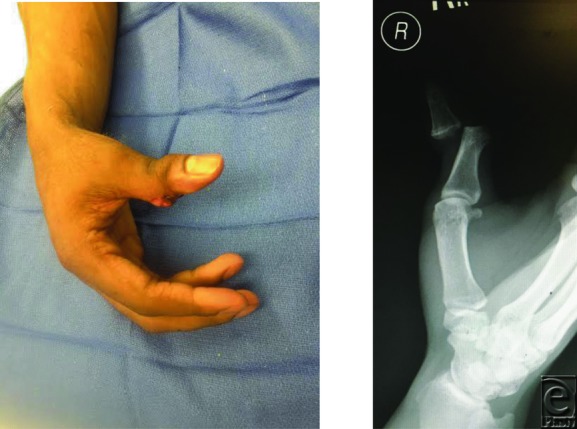


## DESCRIPTION

A 23-year-old man presented to the emergency department with the after being struck in the hand with a basketball. Clinical findings are seen in the image above.

## QUESTIONS

1. **What is the nature of the injury seen above?**
2. **What is the role of radiography in clinical workup?**
3. **What are the common causes of failed reduction attempts in thumb interphalangeal (IP) joint dislocations?**
4. **How are open thumb IP joint dislocations best managed?**

## DISCUSSION

The patient in the image above sustained an open IP joint dislocation of the right thumb. The distal phalanx was dorsally displaced and radially rotated. IP joint dislocations occur following axial load to outstretched fingers. The thumb is a relatively stable joint, and open dislocations of the IP joints are relatively uncommon, with less than a dozen cases reported in the literature.^1^

Radiographs are needed in all hand injuries. Plain radiographs will demonstrate whether a fracture and/or dislocation are present. However, as in this case, radiolucent soft tissue can be displaced into the joint space, impeding closed reduction. In those instances, plain radiographs are not diagnostic. Magnetic resonance imaging could differentiate the soft-tissue injuries but typically is not necessary because open reduction is indicated.

Closed reduction of the IP joint is usually successful, especially with closed injury. The most common reason for failed reduction of the IP joint is avulsion of the volar plate from the proximal phalanx with interposition into the joint.^1^ Salamon and Gelberman^2^ described a case series in which the reduction was blocked by either the volar plate or flexor pollicis longus (FPL) tendon. Other reports indicate it is a combination of the two, or an interposition of the sesamoid bone or osteochondral fragment.^3^^,^^4^

General treatment guidelines of thumb injuries are dependent on the presence or absence of associated fractures, and closed reduction is often initially attempted in the emergency setting. If the patient fails closed reduction, the patient should undergo operative exploration and definitive repair. Structures preventing adequate reduction must be identified and returned to their normal anatomical position. Care must be taken to identify and protect the neurovascular bundles.^1^ An avulsed volar plate can be repaired or extricated from the joint. The joint should then be evaluated for stability by passive range of motion. Arthrodesis of the joint may be performed temporarily with Kirschner wires, and a splint should be applied.

Our patient sustained unsuccessful closed reduction attempts due to the presence of the volar plate and FPL in the joint. He underwent operative intervention, and the volar plate and FPL were returned to their normal anatomical positions. The joint was reduced, the wound was irrigated, and the patient was placed in a splint.
